# The (Mis)Reporting of Male Circumcision Status among Men and Women in Zambia and Swaziland: A Randomized Evaluation of Interview Methods

**DOI:** 10.1371/journal.pone.0036251

**Published:** 2012-05-22

**Authors:** Paul C. Hewett, Nicole Haberland, Lou Apicella, Barbara S. Mensch

**Affiliations:** 1 HIV-AIDS Program, Population Council, Lusaka, Zambia; 2 Poverty, Gender, and Youth Program, Population Council, New York, New York, United States of America; 3 Center for Biomedical Research, Population Council, New York, New York, United States of America; Johns Hopkins University Bloomberg School of Public Health, United States of America

## Abstract

**Background:**

To date, male circumcision prevalence has been estimated using surveys of men self-reporting their circumcision status. HIV prevention trials and observational studies involving female participants also collect data on partners' circumcision status as a risk factor for HIV/STIs. A number of studies indicate that reports of circumcision status may be inaccurate. This study assessed different methods for improving self- and partner reporting of circumcision status.

**Methods/Findings:**

The study was conducted in urban and rural Zambia and urban Swaziland. Men (N = 1264) aged 18–50 and their female partners (N = 1264), and boys (N = 840) aged 13–17 were enrolled. Participants were recruited from HIV counseling and testing sites, health centers, and surrounding communities. The study experimentally assessed methods for improving the reporting of circumcision status, including: a) a simple description of circumcision, b) a detailed description of circumcision, c) an illustration of a circumcised and uncircumcised penis, and d) computerized self-interviewing. Self-reports were compared to visual examination. For men, the error in reporting was largely unidirectional: uncircumcised men more often reported they were circumcised (2–7%), depending on setting. Fewer circumcised men misrepresented their status (0.05–5%). Misreporting by women was significantly higher (11–15%), with the error in both directions. A sizable number of women reported that they did not know their partner's circumcision status (3–8%). Computerized interviewing did not improve accuracy. Providing an illustration, particularly for illiterate participants, significantly improved reporting of circumcision status, decreasing misreporting among illiterate participants from 13% to 10%, although misreporting was not eliminated.

**Conclusions:**

Study results suggest that the prevalence of circumcision may be overestimated in Zambia and Swaziland; the error in reporting is higher among women than among men. Improved reporting when a description or illustration is provided suggests that the source of the error is a lack of understanding of male circumcision.

## Introduction

Randomized controlled trials conducted in Africa have shown that male circumcision (MC) reduces the risk of HIV infection among heterosexual men by about 60 percent [1]–[3]. Based on these findings, a WHO/UNAIDS Technical Consultation recommended that national programs of male circumcision be implemented in settings with low MC prevalence and high HIV prevalence [4]. Estimates of MC prevalence are based on nationally representative household surveys, such as the Demographic and Health Surveys, which rely on self-reported MC status. A growing body of literature, however, suggests that MC self-reports may be inaccurate.

Levels of MC status misreporting have been found to vary depending on the setting and context. For instance, Castellsagué and colleagues [5] pooled data from case-control studies that examined the link between MC and HPV in five countries: Brazil, Thailand, the Philippines, Spain, and Colombia. In the countries in which self-report was confirmed by physical examination performed by a clinician (Brazil, Thailand, and the Philippines), the authors found that self-reported circumcision was accurately reported by 95% of the male participants. The level of inaccurate reporting, however, was greater (7%) among men classified upon examination as circumcised, than the level (2%) among men classified upon examination as uncircumcised.

More recently, Westercamp and colleagues [6] conducted a household-based survey to assess beliefs about male circumcision in Kisumu, Kenya, where MC services were about to be scaled up. Participant circumcision status was determined by visual examination conducted by male interviewers. It is noteworthy that 48% of the men participating in that study refused a visual examination in the household. Of the remaining 52% who consented to the exam, 3.5% of the men classified as circumcised on the basis of the exam misreported their status, while less than 1% percent of men classified as not circumcised reported they were circumcised, overall 98% of those examined correctly reported their circumcision status.

A number of other studies have found substantial discrepancies between self-reports of MC and clinical assessment. Lissouba and colleagues conducted a cross-sectional survey in Orange Farm, South Africa among a random sample of 1,198 men aged 15–49 to examine knowledge, attitudes, and beliefs about MC and to assess the association between MC and HIV [7]. Self-reports of MC status were obtained via face-to-face interview, while circumcision status was determined by a trained male nurse as part of a genital examination. The authors found that 45% of men who had reported they were circumcised were in fact not circumcised according to clinical assessment. The authors attribute a significant amount of misreporting to the confusion between circumcision and traditional initiation rituals in which the foreskin may or may not be removed. The influence of this misreporting on estimates of the association between HIV and MC was found to be substantial. The authors calculate that the adjusted HIV prevalence rate is more than 50% lower among men who were clinically assessed as circumcised (aPR = 7.2%) compared to men with intact foreskins (aPR = 17.2%). There was no difference between HIV incidence and prevalence between uncircumcised men and self-reported circumcised men with a foreskin.

A community randomized trial in Mwanza, Tanzania conducted in 1998–2002, which evaluated the impact of an adolescent sexual health intervention, followed 5,083 adolescent males aged 14–18 and also assessed the reporting of circumcision status at baseline and at 18 months [8]. At each interview, participants were examined by a clinical officer, with no training provided specifically for assessing MC status. The circumcision prevalence was low, at 12%. The study found that at baseline, among males clinically assessed as circumcised, 4.2% reported they were not, while 2.8% of males assessed as uncircumcised reported being circumcised. Across the two rounds of the study, only 79% of the boys who reported being circumcised at baseline reported the same status at follow-up; while 94% of boys who reported being uncircumcised at baseline identified their status as such at follow-up, with some circumcised in the interim. The authors found that only 84% of those boys determined by a clinician at baseline to be circumcised were categorized by a clinician as circumcised at follow-up, indicating that the training and standardization of clinical determination is critical to assessing circumcision status. It also suggests that the variation in the type or completeness of circumcision and in the natural foreskin length may contribute to misclassification [8].

Few studies have assessed the natural variation in the length of foreskin or the completeness of circumcision. In the Chogoria area in the eastern region of Kenya, Brown and colleagues [9] found three general types of circumcision, with variability in the amount of foreskin that had been removed among circumcised men. Urassa and colleagues [10] identified eight out of 202 factory workers as partially circumcised — all of whom had reported that they were circumcised. The Kenyan study among truckers cited above excluded six men from the analysis who were partially circumcised [11]. The population-based study in Kisumu found that among the participants who consented to an exam, nine (3%) were partially or “‘abnormally’ or partially” circumcised [12]. In a study among adolescents in Texas, researchers found that 1.2% of participants were partially circumcised [13]. The importance of distinguishing whether a circumcision is complete or partial is highlighted in a recent study by Maughan-Brown and colleagues who find that partially circumcised men have a 7% greater risk of having HIV than fully circumcised men (p<0.05), and that partial circumcision conferred no protective effect compared to no circumcision [14].

To our knowledge, there have been no quantitative assessments of the accuracy of reports by women of partner circumcision status. The study in Mwanza, Tanzania included qualitative interviews with adolescent girls and found that the majority did not know what circumcision was [8]. Moreover, a clinical study of a microbicide placebo that investigated variance in adherence reporting by interview method in South Africa found that women in a face-to-face interview (FTFI) were significantly less likely than those using audio computer-assisted self-interviewing (ACASI) to report “don't know” when asked about circumcision status of their partners (8% versus 19%). This finding suggests that women may be unwilling to acknowledge that they don't know what is meant by circumcision when asked by an interviewer and thus may misreport partner's MC status in face-to-face interviews [15].

To improve the accuracy of reporting, researchers need to identify and address the underlying reasons for misreporting, including lack of knowledge, misunderstanding the question, translation accuracy, reporting bias, and physical differences in circumcision. To compensate for inadequate knowledge, researchers have recommended the use of visual aids to improve comprehension [8]. Another potential remedy is to describe circumcision to participants. Surveys typically ask only whether the participant is circumcised, assuming the respondent understands what is meant by “circumcision.” For example, the most recent Zambia and Swaziland DHS asked, “Some men are circumcised. Are you circumcised?” [16], [17]. The Zambian Sexual Behavior Survey asked, “Some men or women have been circumcised. Have you been circumcised?” [18].

Social desirability bias may also negatively affect reporting. In settings where MC programs are expanding, respondents may feel increasing pressure to present themselves as circumcised in face-to-face interviews. On the other hand, if circumcision is associated with tribes that have minority or lower status or if MC is perceived as traditional or rustic, circumcision may be underreported. To address the issue of social desirability in surveys, studies in the U.S. and elsewhere have found that the use of computerized self-interviews can significantly improve the accuracy of reporting [19]–[23].

This study was designed to assess how accurately males report their own status and females report that of their partners' in two countries in which MC is scaling up, Zambia and Swaziland. The study also sought to identify and address the potential causes of misreporting and experimentally evaluate methods for improving the reporting of MC status. The analysis that follows focuses on two of the possible reasons for misreporting: (1) lack of understanding, and (2) reporting bias due to social desirability, and finds that misreporting of MC status is largely due to lack of thorough understanding of circumcision.

## Methods

### Ethics Statement

The study was reviewed and approved by the Population Council Institutional Review Board (protocol number 454), the University of Zambia Biomedical Research Ethics Committee (reference number 003-05-09) and the Swaziland Scientific and Ethics Committee of the Ministry of Health and Social Welfare (reference MH/599B). Written informed consent was obtained from all adult participants. For participants under the age of 18, assent was obtained after obtaining written informed consent from the parent/guardian. In accordance with local IRB recommendations, male and female participants were given 50 Lilangeni ($6.25) in Swaziland and 20,000 Kwacha ($4.00) in Zambia as compensation for participation.

### Study design

The study was conducted from July 2009 to May 2010 and was implemented in urban Zambia (Lusaka), urban Swaziland (Mbabane and Matsapha), and rural Zambia (selected wards within 20 kilometers of Lusaka). Men aged 18–50 and their female sexual partners, as well as adolescent boys aged 13–17, were eligible and were recruited from clients visiting HIV counseling and testing (CT) sites, health centers, and from the communities surrounding these clinics. It was not required that couples be married or cohabitate to participate. However, after confirming they were sexual partners, efforts were made to verify the relationship by separately asking each male participant and his female partner a series of questions about the other, e.g., when they first met, how many children they had, what meal they last shared, etc. Interviewers determined the particular questions and how many were necessary to confirm the relationship status of the couple. Couples whose status could not be verified were not permitted to participate; data were not collected on how many couples were not allowed to participate in the study.

A block 6 randomization scheme stratified by site was used to randomly assign participants (1,264 men, 1,264 females, and 840 adolescent males) to one of the three methods of interview. A list of study IDs and random assignment to group was generated for the desired sample size prior to data collection and managed by the site coordinator who assigned participants to study arm in order of intake at enrollment. Each couple in the study was assigned to the same experimental arm; the woman's assignment was based on the man's randomization.

**Figure 1:**
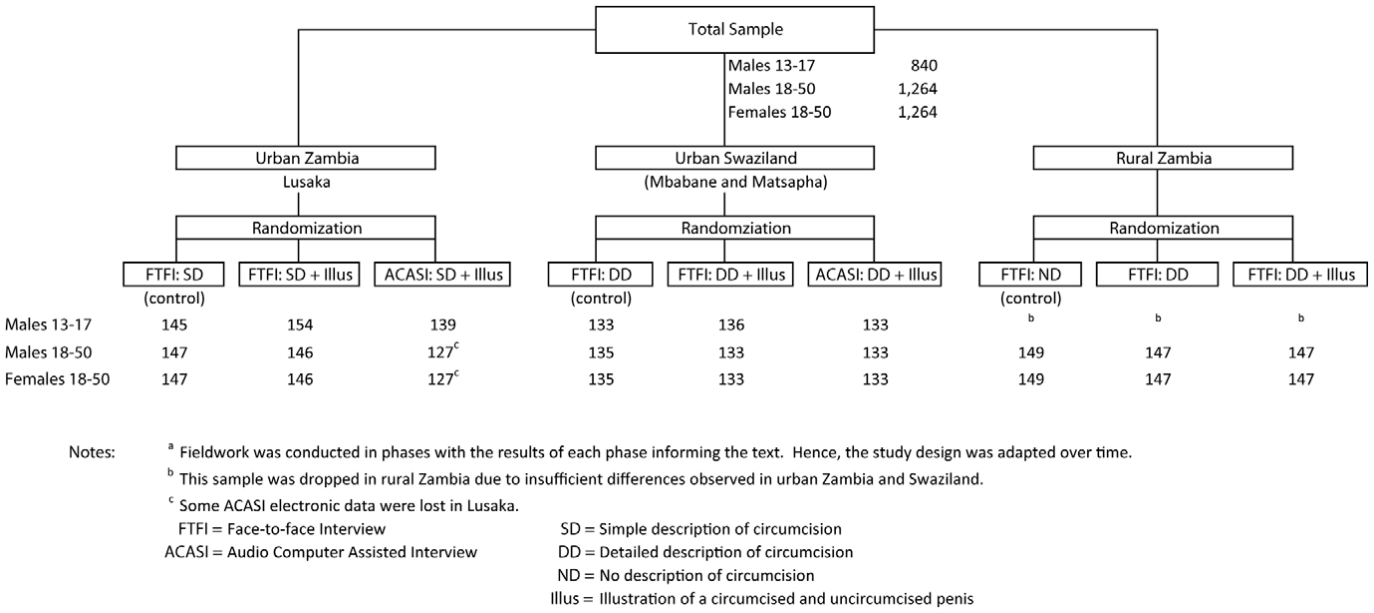
Study design and experimental assignment by setting.

Note that because fieldwork was conducted in phases, with the results of each phase informing the next, the study design was adapted over time and varied by setting. Figure 1 illustrates the study design for each site and analytical sample sizes obtained in each study arm. In each site there were three arms: two different face-to-face interview arms, one of which was a control, and an ACASI arm. In the ACASI arm, respondents answered questions in private and without interviewer assistance, using a touch screen tablet computer. The questions and the MC illustration were displayed on the screen and the questions read to the respondent via prerecorded audio.

With the exception of the control group in rural Zambia where **no description** (ND) of MC was provided, male circumcision was described to each male participant before asking about his circumcision status, and to each female participant before asking about her partner's circumcision status. The descriptions of circumcision were either provided alone or in combination with an illustration. In Lusaka, a **simple description** (SD) was provided: “Male circumcision is when the foreskin of the penis is removed or cut off.” In urban Swaziland and rural Zambia, a **detailed** **description (DD)** was provided: “Male circumcision is the removal of the foreskin from the head of the penis. The foreskin is the skin that covers all or most of the head of the penis of uncircumcised men. You can see if a man is circumcised by looking at his penis when he does not have an erection. When men are circumcised, you can see the head of the penis. When they are uncircumcised, the head may be partially or completely hidden by the foreskin. When the penis of an uncircumcised man is erect (hard), usually the foreskin pulls back and the head of the penis is uncovered.”

**Figure 2:**
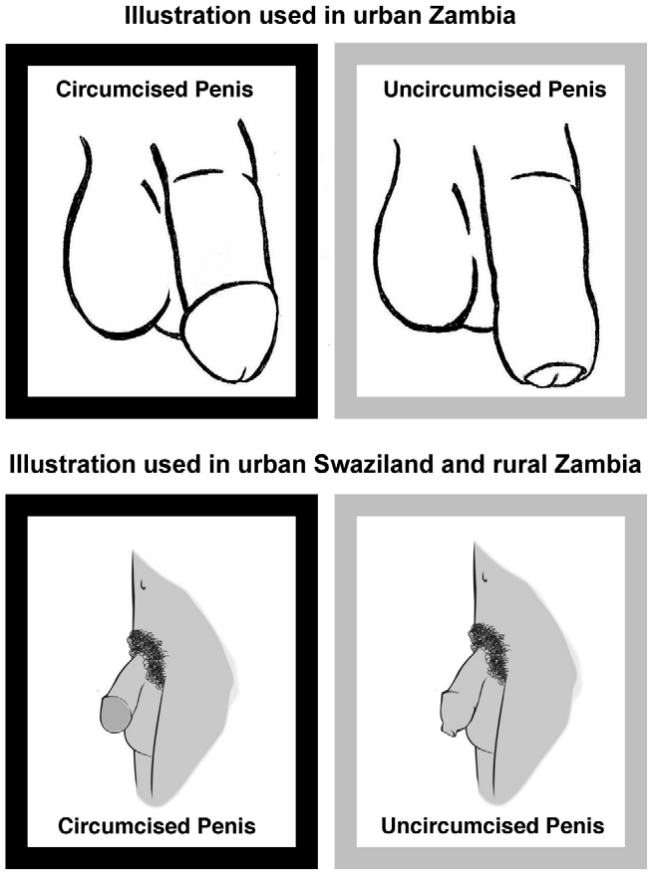
Illustrations of circumcised and uncircumcised penis.

The illustrations used in the study depicted a circumcised and an uncircumcised penis and are shown in Figure 2. The illustration was changed in Swaziland and rural Zambia to improve the quality of the image, as well as to eliminate the possibility that the respondent might be confused because of the slight exposure of the penis glans in the urban Zambia illustration of an uncircumcised penis. It should be noted, however, that the illustration was always coupled with a verbal description of circumcision.

Within the study experimental arms, after the description was read and an illustration shown (if applicable), male participants were asked, “Are you circumcised?” while female participants were asked, “Is the man you came to the clinic with circumcised?” To verify the reported circumcision status of participants, male participants were subsequently asked to undergo a visual examination conducted by a clinical officer or medical doctor who was trained in and had performed MCs in each respective country. Status was categorized as: 1) not circumcised (glans penis completely covered); 2) completely circumcised (glans penis fully exposed); and 3) partially circumcised (glans penis partly covered). In Lusaka and Swaziland, the study and examinations were conducted in a nonclinical HIV VCT site. In rural Zambia, the study and examinations were conducted in district health clinics. To avoid the possibility that prior knowledge of the clinical examination might affect the participant's reporting of his circumcision status, informed consent for the visual examination was requested only after the survey interview was completed.

In order to assess the literacy level of study participants, participants were asked to read a simple sentence. Their ability to read all, part, or none of the sentence was recorded. The sentence used and the method for assessing literacy were based on the approach employed in the Demographic and Health Surveys [16]. In Zambia, the literacy assessment was implemented in English, while in Swaziland participants had a choice between SiSwati and English.

### Statistical methods

Descriptive frequencies were generated by setting and study sample (adolescent male, adult male and female); chi-square tests for differences in proportions tests were used to determine significance when appropriate. Unadjusted and adjusted logistic regression models were used to assess the impact of the experimental method on the misreporting of circumcision status. Odds ratios and adjusted odds ratios are reported for ease of interpretation. A logistic regression on pooled data, combining observations across all sites and sex, was estimated specifically to assess the impact of the illustration on misreporting of MC status. Two different models are presented that assess the impact of the illustration, one was separately run for literate and illiterate participants to provide odds ratios with the conventional interpretation. Another regression model included an interaction term between literacy status and exposure to the illustration to statistically assess the non-linear relationship. This estimation used a computational approach suggested by Norton et al. [24] and a method for interpreting the estimation results based on the odds of misreporting [25]. All estimated standard errors in the regression analyses were adjusted for clustering by interview method. Participants who reported that they did not know their MC status were not included in the analysis of misreporting.

## Results

### Descriptive analysis

The characteristics of the participants are presented by study site in Table 1. In urban Zambia, 12% of adolescent boys and 21% of adult men were classified as circumcised. Circumcision prevalence is similar in urban Swaziland, but lower for rural Zambia. The prevalence of MC in urban areas of each country is somewhat higher than MC prevalence found by the DHS for each country [16]:p.214, [17]:p.176], and likely reflects the fact that national programs of MC scale-up started in these urban areas in each country in early 2009. Few men were categorized by the clinician as partially circumcised in urban Zambia and Swaziland (<2%). A higher number and percentage of men 22/443 (5%) were classified as such in rural Zambia. This result, however, may be explained by the fact that a clinical officer who joined the study in rural Zambia was more inclined to classify men as partially circumcised (15 of the 22 partial MCs are attributable to this clinician). Although it is not possible to confirm whether the designation by the clinical officer was in error, differential classification of circumcision status among clinicians is not uncommon [8], [26]. These cases were removed from subsequent analysis.

**Table 1:** Demographic characteristics of study participants (percentages unless otherwise indicated).

	Urban Zambia			Urban Swaziland			Rural Zambia^a^	
Characteristic	Males 13–17 N = 438	Males 18–50 N = 420	Females 18–50 N = 420	Males 13–17 N = 402	Males 18–50 N = 401	Females 18–50 N = 401	Males 18–50 N = 443	Females 18–50 N = 443
**Circumcision Status (clinician assessment)**								
Not circumcised	88	77	—	86	78	—	86	—
Fully circumcised	12	21	—	13	20	—	9	—
Partially circumcised	<1	1	—	1	2	—	5	—
Don't know (self-report)^b^	3	1	8	<1	<1	5	<1	3
**Mean age in years**	15.1	34.6	29.1	16.1	29.7	25.4	39.9	33.2
**Highest level education completed**								
None	3	2	5	2	2	1	3	11
Some/compl. primary	47	19	34	28	11	15	37	50
Some/compl. secondary	48	52	45	67	59	60	48	38
Higher	2	27	16	3	29	25	12	1
**Marital status**								
Never married	97	17	15	99	37	49	0	.5
Currently married or living together	3	81	82	1	62	51	100	99
Separated, divorced, widowed, other	0	3	3	0	1	0	0	.5
**Tribe**								
Ngoni	11	8	8	—	—	—	9	5
Tonga	11	10	11	—	—	—	35	26
Bemba	25	29	29	—	—	—	12	8
Swazi	—	—	—	100	98	98	—	—
Other	53	52	52	0	2	2	44	61
**Religion**								
Christian	99	97	99	95	95	99	100	100
Other	1	3	1	5	5	1	0	0
**Literacy test results**								
Cannot read	13	6	19	4	4	2	16	32
Able to read only part of sentence	16	5	14	1	1	3	7	15
Able to read whole sentence	71	89	67	94	95	95	77	53
**Previously heard of MC**	73	90	82	93	96	93	90	88
**Comprehensive knowledge of HIV prevention** c	33	62	53	66	76	77	82	67
**Ever use of condoms**	12	92	82	21	94	94	83	68

a Adolescent sample was dropped in rural Zambia due to insufficient differences observed in urban Zambia and Swaziland.

b Don't know and partially circumcised cases dropped from analysis of misreporting of MC status.

c Defined as answering correctly that reducing partners, using condoms, and abstinence are HIV prevention methods; that a person cannot become infected by sharing food with someone who has AIDS; and that it is possible for a person who looks healthy to have AIDS [16].

Although adolescents in Lusaka were slightly more likely to report not knowing their MC status, most adult men in these settings provided a response to the question of circumcision status. That said, the prevalence of “don't know” responses among men and boys in Lusaka was significantly higher in ACASI interviews than in FTFIs (5% versus <1%, p<.01), likely because respondents were less willing to reveal ignorance of status in the FTFIs. Women were much more likely to report that they did not know the MC status of their partner in all three settings. Further, “don't know” responses were significantly more prevalent in ACASI than in FTFIs in urban Swaziland (9% versus 3%, p<.01) and rural Zambia (6% versus 2%, p<.05)); for Lusaka, the data are directionally consistent but not significant.

The mean age of the adolescent sample was 15.1 years in Lusaka and 16.1 years in Swaziland. For adult men, the mean ages were 34.6, 29.7, and 39.9 in urban Zambia, urban Swaziland, and rural Zambia, respectively. The mean ages for women were 29.1, 25.4, and 33.2. As expected, the adult sample was slightly more educated than the adolescent sample, since a number of adolescent males were still attending school and had not completed their education. Males had higher levels of education than females in Zambia, with greater educational parity between the sexes in Swaziland. Almost all adolescent males were unmarried. In both urban Zambia and Swaziland, more than half of the couples were in formal unions; a substantial percentage of discordant reporting of formal union status was observed between males and females in Swaziland. Tribal affiliation varied in Zambia, reflecting the ethnic diversity within Lusaka district. Almost all participants indicated their religious affiliation as Christian in both countries. The ability to read a simple sentence in English was moderately high among males in Zambia, but substantially lower for women, particularly in rural areas. In Swaziland, literacy was nearly universal, but in that country respondents were allowed to choose to read either a sentence in SiSwati or in English for the literacy evaluation, which may account for the higher rates. Self-reported knowledge of MC was quite high in both countries, although relatively lower among adolescent males and among females in urban Zambia. Demonstrated comprehensive knowledge of HIV was higher than that observed in the Zambian 2007 DHS or the Swaziland 2006 DHS, but it is not possible to ascertain if this is due to trends in the indicator or the differences in the representativeness of the samples. Finally, ever use of condoms was high for adults in both countries, although relatively lower in rural Zambia, while adolescents had a substantially lower prevalence of condom use.

As seen in Table 2, 29% of adolescent males and 24% of adult males declined the visual examination in Lusaka. Refusals were substantially lower in Swaziland and rural Zambia. Refusal rates in Lusaka were unrelated to interview mode or to most demographic characteristics, with the exception that participants with a secondary or higher education were more likely to refuse the exam (p<.05, data not shown). In Swaziland, participants who were unable to read English were significantly more likely to refuse an examination (p<.01, data not shown).

**Table 2:** Visual examination refusal rates.

	Males 13–17			Males 18–50		
	Interviewed	Examined	Refused Exam	Interviewed	Examined	Refused Exam
**Urban Zambia**	438	311	29%	420	318	24%
**Urban Swaziland**	402	381	5%	401	371	8%
**Rural Zambia**	—	—	—	443	439	1%

**Figure 3:**
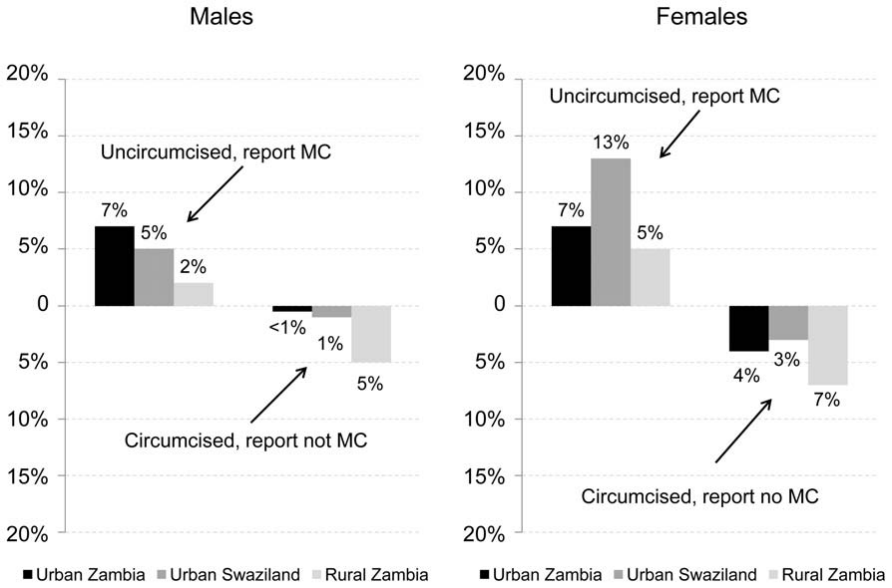
Direction of misreporting of MC status among those reporting.

As can be observed in Figure 3, for men in urban Zambia and Swaziland, the direction of misreporting of MC status is unidirectional — uncircumcised men more often report that they are circumcised: 7% in Lusaka and 5% in Swaziland. In rural Zambia, 2% of uncircumcised men reported they were circumcised and 5% of men classified as circumcised reported they were not circumcised. As suggested previously, this latter result was potentially attributable to a new study clinician who was more apt to classify cases as partial circumcisions; it is unclear whether these cases were misclassified, incomplete circumcisions, or naturally occurring shorter foreskins. With these cases removed from the total, only 2% of the circumcised men in rural Zambia report that they are uncircumcised, rates similar to those found at the other study sites.

The figure further illustrates that misreporting among women is significantly higher than among men and runs in both directions. The highest misreporting (13%) was found among Swazi women who have uncircumcised partners. Further, as indicated in Table 1, a nontrivial proportion of women report they do not know their partner's status (3% in rural Zambia, 5% in Swaziland and 8% in urban Zambia). Overall, these findings reveal a considerable degree of misunderstanding among women about the circumcision status of their partners.

### Multivariate analysis

To assess whether introducing different methods of describing and illustrating MC significantly reduced the prevalence of misreporting after adjusting for potential confounding factors, logistic regression models were estimated. The dependent variable was coded 1 if the participant misreported circumcision status (in either direction) and 0 if reported circumcision status was consistent with the clinician's assessment. All characteristics shown in Table 1 were included in the adjusted logistic estimations. The results of the regressions for each experimental arm by study site are displayed in Table 3; the effects of the model covariates are discussed below, but the odds ratios for covariates are not shown in the table. The similarity between the unadjusted and adjusted odds ratios suggests that the randomization was largely effective in ensuring independence between the participant characteristics and interview mode.

**Table 3:** Logistic regression assessing experimental arms on misreporting of MC.

	Males		Females	
	OR	AOR	OR	AOR
**Urban Zambia**	N = 615	N = 591	N = 293	N = 284
FTFI Simple Description (Ref)	1.0	1.0	1.0	1.0
FTFI SD + Illustration	1.3 (.53–3.4)	1.5* (1.1–2.1)	.91 (.38–2.2)	.83 (.62–1.1)
ACASI SD + Illustration	1.7 (.69–4.3)	1.8** (1.7–2.0)	.94 (.38–2.4)	.79 (.51–1.2)
**Urban Swaziland**	N = 734	N = 700	N = 343	N = 332
FTFI Detailed Description (Ref)	1.0	1.0	1.0	1.0
FTFI DD + Illustration	.98 (.40–2.4)	1.0 (.96–1.1)	.81 (.39–1.6)	.80** (.73–.87)
ACASI DD + Illustration	2.3* (1.1–5.0)	2.3** (2.0–2.5)	1.1 (.52–2.2)	1.1 (.80–1.5)
**Rural Zambia** ±	N = 416	N = 402	N = 403	N = 398
FTFI No description (Ref)	1.0	1.0	1.0	1.0
FTFI Detailed Descript	1.0 (.20–5.2)	.61** (.48–.78)	.77 (.33–1.8)	.63** (.54–.73)
FTFI DD + Illustration	1.3 (.30–6.1)	1.1 (.68–1.6)	.78 (.33–1.9)	.91 (.82–1.02)

† p<.10,

* p<.05,

** p<.01.

**OR:** odds ratio; **AOR:** adjusted odds ratio: adjusted for all demographic and other variables in Table 1; significant covariates discussed in text. Standard errors adjusted for clustering within interview method for. Samples sizes based only on those participating in the visual examination.

**Ref:** reference or base category.

± Dropped cases in which clinician indicated partial circumcision (n = 44) – see text.

The unadjusted and adjusted odds ratios presented in Table 3 indicate that for urban Zambia and Swaziland the illustration in either the FTFI or the ACASI interview improved the reporting of MC status. In fact, for urban Zambia, the experimental arms indicate significantly higher misreporting among men. In Swaziland, among males the results in the ACASI and illustration arm suggest that the odds of misreporting were more than double when ACASI was used. For women in Swaziland, the illustration, when used in the context of a FTFI, significantly reduced misreporting when compared to the detailed description alone. In rural Zambia — the only setting in which a detailed description and illustration were compared with current practice in household based surveys, which involves no description — male and female participants who received a more detailed description of circumcision (with no illustration) had significantly lower odds of misreporting when other factors were controlled and standard errors adjusted for clustering within experimental arm. Reductions in misreporting, however, were not apparent among males in rural Zambia who received both a detailed description and an illustration. For females in rural Zambia, the addition of the illustration improved reporting compared to no description, but not significantly.

Few demographic or other characteristics were found to be consistently significantly associated with misreporting: being older lowered the misreporting for males in both urban and rural Zambia (p<.05), while illiteracy increased misreporting (p<.01) among females in rural Zambia; also, being married increased the odds of misreporting (p<.01) among females in Lusaka (data otherwise not shown).

Although the tools tested in the different experimental arms did not consistently reduce misreporting, one additional step was undertaken to determine whether the illustration decoupled from the interview method reduced misreporting, particularly for those who were not able to read a simple sentence. To investigate this question, the data were combined across all sites and by sex, and two regression models were estimated with study site and sex included as covariates. The first model estimates the impact of the illustration separately for literate and illiterate participants (top panel of Table 4). This approach allows for the presentation of odds ratios (OR) that have the conventional interpretation. To assess the statistical significance of the interaction between literacy and the illustration, a second logistic regression model was estimated (bottom panel of Table 4). Unlike linear models in which the interaction term reflects the change in the effect of one explanatory variable on the outcome for a unit change in the other, in nonlinear models the marginal effect cannot be similarly computed or interpreted [24], [25]. To interpret the results, the odds of misreporting for each combination of the interaction are provided [25].

**Table 4:** Logistic regression on pooled data assessing illustration on misreporting of MC status.

Model 1: Separate model	Literate Participants		Illiterate Participants	
	OR (N = 2226)	AOR (N = 2197)	OR (N = 544)	AOR (N = 538)
**Illustration of MC provided**	1.0 (.65–1.6)	1.1** (1.0–1.1)	.66 (.32–1.3)	.62** (.52–.72)
ACASI	1.5† (.98–2.2)	1.4** (.20–3.1)	1.2 (.55–2.5)	1.3† (.96–1.7)
Study site: Urban Zambia		.52** (.36–.75)		2.3 (.32–16.3)
Study site: Rural Zambia		.28** (.25–.31)		2.1† (.92–4.8)
Female		2.5** (1.9–3.4)		4.6** (2.3–9.0)
Age (continuous)		.98† (.95–1.0)		.97** (.96–.97)
Attended primary or lower		1.2 (.48–3.0)		.84 (.31–2.2)
Married or living with partner		1.3 (.78–2.2)		.42** (.23–.79)
Comprehensive HIV Knowledge		.82 (.49–1.4)		.77† (.57–1.1)
Ever heard of MC		.67 (.39–1.2)		1.5 (.78–2.7)
Ever used condom		1.2 (.95–1.5)		1.4 (.78–2.7)

Model 2: Interaction Model±	Coefficient	SE	p-value
Interaction: Illiterate * Illustration	−.045 (−.12–−.01)	.01	p<.01
**Odds of Misreporting** ±			
Illiterate and No Illustration	.13		
Illiterate and Illustration	.10		
Literate and No Illustration	.06		
Literate and Illustration	.08		

† p<.10,

* p<.05,

** p<.01;

**OR:** odds ratio; **AOR:** adjusted odds ratio and significance tests. Tribal affiliation included in model, but results not shown. Standard errors adjusted for clustering within interview mode. Models do not include cases of partial circumcision.

± Statistical computation based on approach by Norton et al. [24]; includes full set of covariates shown above (results not shown). Odds of misreporting based on estimation approach suggested by Buis [25].

As seen in the top panel of Table 4, the effect of the illustration differs for literate and illiterate participants. In the unadjusted model, the ORs of the impact of the illustration are not significant and reveal no differences relative to the reference group of no illustration for either literate or illiterate participants. The adjusted odds ratio (AOR), however, when covariates are included and standard errors are adjusted for clustering within interview method, indicates that the illustration significantly reduces misreporting among illiterate participants. The counterintuitive and slight increase in misreporting by literate participants with the addition of an illustration (a marginal increase of 10% on the odds of misreporting) was initially puzzling. However, the original illustration used in Lusaka was subsequently thought insufficiently clear, since part of the glans was revealed in the uncircumcised penis. When the Lusaka data are excluded from the analysis, the alternate illustration significantly reduces misreporting among both illiterate and literate participants (data not shown). For illiterate participants, the illustration reduces the odds of misreporting by 34% in the OR and by 38% in the AOR models, with the result significant only in the adjusted estimation. The effect of the illustration by literacy status is confirmed in the model that reveals a significant interaction term at p<.01 (bottom panel of Table 4). Further, the odds of misreporting for each sub-category of participant indicate that the greatest difference in the prevalence of misreporting is between illiterate and literate participants when no illustration is provided, an illustration reducing by 3% the misreporting by illiterate participants.

The top panel of Table 4 reveals some additional factors associated with misreporting of circumcision status. For instance, participants surveyed by ACASI have significantly greater odds of misreporting their MC status than do those interviewed face-to-face (as also indicated in Table 3 for male participants). Further, literate females have 2.5 times the odds and illiterate females over 4.5 times the odds of misreporting relative to males. Study participants in Zambia who are literate, older participants, and illiterate participants who are married or living with their partner, are less likely to misreport. Interestingly, knowledge of HIV prevention methods, prior awareness of MC, and ever having used a condom do not generate consistent or significant effects, although comprehensive knowledge marginally reduces the odds of misreporting among illiterate participants (p<.10)

## Discussion

The primary objective of this study was to provide evidence-based recommendations for the collection of self-reported data on MC status to researchers and program managers interested in measuring the prevalence of male circumcision in a general or study population. It also sought to inform HIV prevention trials and observational studies involving female participants, which rely on women to identify the circumcision status of their partners. The study assessed various tools for improving the reporting of circumcision status, including a) a simple and a more detailed description of male circumcision, b) illustrations of a circumcised and an uncircumcised penis, and c) computerized self-interviewing technology. Reporting of MC status was validated by visual examination.

A high participant refusal rate for visual examination of circumcision status occurred in the Lusaka study site. The high refusal rate for Lusaka suggests that visual examinations to validate self-reporting of MC status may be difficult to implement in some settings, replicating similar findings elsewhere [6]. One possible explanation for the high number of refusals is that participants may have been uncomfortable with the visual examination in a nonclinical, HIV VCT setting, despite the use of a private room.

Between 2 and 7% of males in the study misreported their circumcision status according to the clinical exam. For males the error in reporting of MC status is largely unidirectional, with uncircumcised men reporting that they are circumcised; few circumcised men misrepresented their MC status. The results of this study suggest that national estimates likely overstate actual MC prevalence. Further, in assessments of the influence of MC on HIV incidence, estimates of the impact of MC are likely to be attenuated given misreporting of MC status. These results demonstrate that inaccurate self-reports of MC status are a concern in Zambia and Swaziland, paralleling findings from other countries (e.g., Weiss et al. [8], Urassa et al. [10], Risser et al. [13], Schlossberger et al. [27], and Thomas et al. [28]).

Between 11 and 15% of women inaccurately report the circumcision status of their partners, with the error in reporting in both directions. Clinical trials testing potential HIV prevention technologies and behavioral interventions using partner's MC status to control for confounding may be inaccurate if measurement error in MC status is correlated with the misreporting of other self-reported indicators, e.g., adherence to product use in clinical trials, socioeconomic status, and sexual or other risk behaviors (alcohol and drug use) [29].

The study results indicate that audio computer-assisted self-interviewing (ACASI) did not improve, and likely compromised, the self-reporting of MC status. The poor performance of ACASI suggests that participants felt a greater obligation to respond honestly to an interviewer, implying that social desirability bias was probably not a factor in misreporting circumcision. As MC programs are scaled-up and mass media messaging becomes pervasive, social desirability bias may become more pronounced. The face-to-face interviews also likely provided a greater opportunity for the interviewer and participant to discuss the meaning of male circumcision.

The study found that providing an illustration for illiterate participants improved reporting of MC status: misreporting among illiterate participants declined from 13% without an illustration to 10% when one was provided. Counterintuitive results indicate that misreporting was slightly more common among literate participants when they were given an illustration; although the higher level of misreporting was not as substantial. Moreover, this anomaly disappears when the data from urban Zambia—where a potentially ambiguous illustration was used—are dropped from the analysis. The overall conclusion to be drawn is that for studies that rely on self-reports of MC status detailed descriptions and/or illustrations provide a useful method for improving the reporting of MC status by both males and females, but should be pilot-tested for appropriateness. Note, while this should improve reporting, it will not eliminate misreports of MC status.

There are limitations to this study that should be considered when interpreting results. A key concern is that the sample is not representative of the Zambian and Swazi populations, and therefore caution is needed in extrapolating the data to prevalence estimates of MC in each country. A second consideration is that as MC programs scale up and messages about the benefits of MC reach a larger proportion of people, there may be changes to misreporting: on the one hand, a potential decrease in misreporting resulting from poor comprehension; on the other hand, a potential increase in misreporting because of increased social desirability bias. A final limitation is that the study did not directly address the issue of partial circumcision. Partial circumcisions were rarely observed in the study; however, one of the study clinicians classified circumcisions as partial more often than the other clinicians did. Since only complete circumcisions are thought to effectively reduce HIV infection for men, more research needs to be done to understand the implications of variations in foreskin length. As Weiss [8] suggests, perhaps circumcision status should be classified by foreskin length, rather than as a dichotomous indicator.
